# Abstract of the Proceedings of the Buffalo Medical Association. Feb. 2d and 9th, 1875

**Published:** 1875-03

**Authors:** W. W. Miner

**Affiliations:** Secretary pro tem.


ART. II.—*Abstract of the Proceedings of the Buffalo Medical Association at the Meeting held February 2d,* 1875. Reported by W. W. MINER, M. D., Secretary pro tem.

The President, Dr. JAMES P. WHITE, called the meeting to order. The members present were Drs. J. F. Miner, White, Potter, Harding, Fowler, Boysen and Miner. Dr. W. W. Miner, was chosen Secretary pro tem.

Dr. P. W. Van Peyma, was elected a member of the Association. The report of committee on the matter of providing for the warming, lighting and janitor's care of the room, was presented and laid on the table.

The President then called on the members for voluntary contributions.

Dr. J. F. MINER, remarked that a subject of great general and public interest, at present, is that of the prevalence of epidemic disease. It is now three years since there prevailed here to a great extent, Cerebro-Spinal Meningitis. No other such fearful epidemic as this was, has visited us; none so terrible, so fatal in effects. It lasted a few months and passed away. Its nature, presence here and disappearance is still an unexplained mystery. About two years since there occurred an epidemic among horses; scarcely a horse escaped the disease; a similar disease, later and in milder degree, affected men to a certain extent. After prevailing here a short time, it having swept through the entire country, disappeared in a manner as mysterious as was its coming. This year and for some time past Scarlet Fever has been epidemic in this city; has been severe in character. The various points having reference to the disease have been discussed in the papers, by all denominations of physicians and by the community, still we are as much in the dark as ever concerning the origin and character of the epidemic; no new facts have been established; we know of nothing that will prevent the extension of the disease or shorten its course where it has begun. We know that mild cases generally continue thus to their termination, and that those cases which are fatal give indication of this character at the commencement of the disease. A very capable physician who has had a wide experience here during this scarlet fever epidemic, says, that one out of five of his cases has been fatal. His treatment I have no doubt has been as judicious, discriminating and appropriate as that of any practitioner in the city. He shows a degree of frankness and an adherence to truth that I admire, since there are so many who would not acknowledge the facts thus unreservedly. My own experience has been more limited, and most of the cases I have been called to I visited in consultation with others and for the reason that they were going to end fatally. The statistics of the Health Physician show that the disease has prevailed to a great extent and very fatally. It would be very satisfactory if we could trace the causes of this epidemic to sewers, drains, cellars and low places, but it has occurred in the high parts of the city. Those of us who have practiced in the country, know that the disease prevails upon the tops of the highest hills. Its period of incubation is not definite. Other diseases whose infectious character we are sure of have a fixed period, as measles, small pox, syphilis, etc. The public examination into the character of this disease has determined upon no really preventive means or method; in fact the course of the disease is not influenced by remedies except as small pox, typhoid fever, etc., are.

The question has come to me this evening from the parents of a child sick with scarlet fever:—"Is it any safe-guard to send the other children into the country?" I think it well to take all practicable means for preventing the spread of the disease. Friends will feel better if they protect themselves as much as they can, and if fatal results occur they have less misgivings. I advised separation in this case. Do not feel that it will ensure those thus isolated from the disease; very frequently several children of a family escape, though others with them go through the course of the disease. Possibly others are more satisfied than myself on this question of prevention and origin.

Dr. T. F. BOYSEN said he had just read in the German of Ziemssen's Cyclopædia of Practice, Dr. Thomas' article on Scarlet Fever, whose opinion there expressed was that the disease is always communicated from one to another, and that it did not arise spontaneously or sporadically, though the patient may appear to have been entirely isolated from infection. He believes in the (leib contagium) germ theory, says there is a distinct microscopical germ peculiar to the disease, that the single germs or cells are found joined together, forming chains of cells. He presents cases illustrating the transfer of the germs in clothing, and says it is carried in milk and in other ways.

Dr. POTTER remarked that however uncertain other points connected with the subject may be, he thought that the matter of the disease being a blood poison was settled, and that it was capable of being communicated not only by the inoculation of blood, but by means of other secretions. We cannot always explain the means of infection in particular instances, but it is not infrequent for the disease to affect all the children of a family, and the fact that sometimes it does not, does not argue against its contagiousness. Circumstances may render one at times susceptible to infection when at other times this susceptibility does not exist. The infection theory furnishes very satisfactory explanation in general for the spread of the disease.

Dr. MINER—I would not deny the infection theory. Most all authors sustain it. There must, however, be a reason why our children this year have scarlet fever outside of infection. Should grant the infection theory but it does not explain the several years' absence of the disease which sometimes occurs. The theory of infection is not sufficient, there are other and general reasons why it prevails now; is then again several years absent; is at times mild in character, then fatal. This variation exists not only in one locality, but through the country and everywhere. Further, we may safely predict that scarlet fever will again disappear from our city, while all the local imperfect hygienic influences are as operative as now, and while the conditions of infection are unchanged, so that whatever may be the influence of infection in extending the disease, it certainly is not the sole cause of its prevalence.

Dr. LUCIEN HOWE wished to remark not so much upon the origin of scarlet fever as upon some of its sequelæ. Dr. E. H. Clark, of Boston, has advocated the importance of proper treatment of the affections of the ear which follow this disease. Dr. Roosa, of New York, also says that many cases come to him showing the unhappy results which sometimes follow inattention by the physician to the ear troubles connected with scarlet fever. These sequelæ usually take the form of an acute suppuration of the external or middle ear. The former is comparatively harmless. It is readily recognized by the swelling, deep seated pain and tenderness of the external canal with the abundant and often watery secretion. The best treatment for this is a douche of warm water, or mild astringent solutions. The latter condition is dangerous as resulting frequently in permanent deafness. The inflammation reaching the middle ear from the throat through the eustachian tube gives rise to pain, but no tenderness; a small amount of thick pus is secreted, while the membrana tympani is swollen, and without the bright reflex. For treatment the warm douche may be used or leeches applied to the tragus, which is much the most suitable place for their application generally in affections of the ear; if closure of the eustachian tube has occurred, pus is confined in a closed cavity, and the surgical indication is plainly to give it vent. Puncture of the membrana tympani was formerly a very much opposed proceeding; it is the proper treatment of these cases. A knife-shaped probe, designed for the purpose, may be used, or in its absence, puncture may be made with a large needle. If the natural process of evacuation, by ulceration and perforation of the membrana, occurs, it leaves a ragged opening which is not apt to close again, and a condition of deafness which is but poorly remedied by an. artificial tympanum. If this bursting does not occur the pus hardens in the middle ear and greatly obstructs the action of the chain of bones. In short, deafness is the natural result of these conditions.

Dr. HARDING remarked that he has treated more cases during the last month than previously, and had been less fortunate than the physician quoted. In a family which he cared for, the first one affected by scarlet fever died thirty-six hours after attack, another in three days, another in thirty-three hours, and one in another family occupying the same house, an adult, died after two weeks illness. He had used sulpho-carbolate of soda in this affection, and found in one instance it reduced the pulse twenty beats, twelve hours after commencing its use.

Dr. White said that this subject had engaged attention considerably and to an unusual extent of late. He had watched epidemics for the last forty years, and did not regard this epidemic as much more fatal than others. His personal experience during this epidemic had been limited, though he had visited in consultation a considerable number of cases that were of fatal character, should not have been called to these except for their serious nature. Desired to say that he had not considered this epidemic more malignant in character than some former ones had been. In one of these epidemics he recalled instances where all the children of a family, and even all the family had been swept away by the disease. He considered escape from an epidemic with a fatality of fifty per cent fortunate, if the type of the disease was really the malignant one.

Scarlet fever prevails endemically as does measles and other like diseases. It is a communicable disease, and those who are exposed constantly to it in the nursery are much more likely to have it than those not thus exposed to scarlatinal emanations. It is not as communicable as are some other infectious diseases, and the period during which a patient is capable of transmitting the disease to others is of uncertain duration. It is much safer to say that the disease is in some way communicable from one affected by it to another, from the sick to the well, than to simply say that we are ignorant of its causes and of its method of extension. The prognosis in this disease depends very much upon the character presented in its inception. Cases are frequently hopeless from the beginning just as they often are in cholera, puerperal fever, etc. I would not say that it is not more subject to control than might be inferred from the remarks made to-night. Where do these epidemics of scarlet fever come from? They come from where cholera comes, from defective drains, sewers, undrained cellars, hot, crowded apartments, fomites, etc. I would not say that these alone are sufficient to give rise to the disease, but these conditions render persons more susceptible to infection by the disease than they would otherwise be. In my own experience both as attending and consulting physician I have found these cases oftener where the hygienic conditions were favorable to disease or were such as to render a susceptibility thereto. Again, conditions favorable to disease may sometimes exist where the location of a dwelling, its construction and appurtenances are supposed to be faultless, and these conditions are found only after disease itself has instigated careful searching out of hidden causes. I am of the opinion that defective hygienic conditions determine the prevalence of this epidemic to a certain extent, and think that in the quarters of the city where the conditions are more favorable to health there has been very slight fatality from the disease. I would therefore urge the importance of proper sanitary regulations or of hygienic measures in preventing the extension and severity of the disease.

After some farther discussion as to the prevalence of the disease in particular localities and its severity there, participated in by Drs. Potter, Miner and White, it was voted that the subject of scarlet fever be resumed at a special meeting appointed for one week thereafter.

---

*Special Meeting Thursday Evening, February 9th*, 1875. Reported by E. N. Brush, M. D., Secretary, pro tem.

Dr. James P. White, President, in the chair. Members present Drs. White, Rochester, J. Cronyn, Wyckoff, Shaw, Gay, O'Brien, W. W. Miner, Harrington, Chace, Greene, Samo, Fowler, Lynde, Little and E. N. Brush; by invitation Dr. Elliott, E. B. Potter and W. Cronyn U. S. N.

On motion of Dr. Wyckoff, Dr. E. N. Brush was elected Secretary pro tem.

Dr. White announced the object of the meeting, and referred to the prevalent excitement concerning Scarlet Fever. He was sorry that a more general invitation had not been extended to the members, but hoped that those present would discuss the subject freely. He said that physicians owed a duty to the public in an emergency like this; that they were expected to furnish advice for the prevention as well as the cure of disease, and the community have a right to look to this association for guidance in this as well as all similar emergencies. The questions to be considered are: Is the disease prevailing as an epidemic? Is it unusually malignant in character? How is it propagated? Is it communicable? and what means should be taken to prevent its spread?

This association is called upon to define the hygienic and sanitary measures to be pursued in procuring its arrest. We will be asked, shall the patients be isolated, shall children from families in which the disease is present be kept out of the streets and schools, how long before it is safe for our children to return to school? We are here also to consider in a general way what is the efficacy of remedies in the disease. What course of treatment in this epidemic has been attended with success. All these points should be discussed in a dispassionate manner and care be taken that differences of opinion are not allowed to seem greater than they really are. In regard to the care of the patient during convalescence something should be said, as often disastrous consequences ensue from undue and ill-advised treatment.

Dr. ROCHESTER remarked that there were many theories as to the source of scarlatina, but he was never satisfied that they were more than theories. An attempt has been made in England and in other countries to ascribe its prevalence to certain conditions of moisture, he did not believe that it arose *de novo* from these causes, but that it was materially influenced by them; its origin as the origin of measles and small pox is unknown. We only know that it is a disease of youth, exceptionally attacking adults. He believed strongly in the doctrine of susceptibility, also believed and did not hesitate to express the opinion that it was communicable, that it could be carried by clothing, skin, etc., from one person to another, but that it required long continuation in the room to be able to carry the poison. As an example he mentioned a case showing that it could be carried by a nurse. A lady acquaintance of Mrs. Dr. Hunt, had been in Mendon, Ont., county, nursing a case of scarlatina, she came to Buffalo to rest, at a time during which there was not known to be a case of scarlatina in the city.

This lady wore in Mendon, a woolen dress, which upon leaving the place she packed in her trunk where it remained until she reached Prof. Hunt's, when she again wore it daily. A short time after entering Prof. Hunt's family his daughter was taken sick with scarlatina and died, not another case occurring in the vicinity nor in the city as far as known.

The tenacity with which the poison clings to clothing, articles of furniture, etc., is remarkable. Children in the family where it is present, often escape, but it is due to lack of exposure, want of susceptibility or other accidental cause. As to public schools, inquiry will show that they are the means of propogating the disease to a remarkable degree. The schools are crowded and badly ventilated, and are frequented by children in whose families scarlatina is present. We had often known of instances where children were sent to school from such families after he had forbidden it.

Therapeutically, common sense and good judgment were the best guides. One child may require quinine, iron, ammonia, etc., another local treatment, and a third simply a saline cathartic and Dovers powder. A daily change of bed clothing and the rainment of the patient should be insisted on, a plentiful supply of fresh air to the sick room should also be provided for.

The use of baths and the employment of other agents to reduce the fever were also essential in some cases.

All other children should be rigorously excluded from the sick room, the patient should remain isolated until desquamation had ceased. The speaker did not believe that enough was known concerning disinfectants to allow us to arrive at any definite conclusions regarding their action, cleanliness and fresh air seem to be the best disinfectants. The period of contagion was uncertain, but he believed that the child could impart the disease at any time during its course. The appearance of the sequelæ should be watched as the worst features were liable to follow the mildest cases. Nephritic disease often was an attempt on the part of the system to throw off the poison which had not been properly eliminated by the natural course of the disease.

Diptheritic complications are always serious, they seem to find in this condition a suitable soil upon which to work. He did not, however, believe in the identity of the two. Had lost two children in a family who had previously passed safely through scarlatina. Did not believe that the present epidemic was more serious than some which had visited the city in former years, thought that it was more prevalent but not unusually fatal. In 1854 an epidemic occurred in this city in which he saw a large number of cases with the late Drs. Wilcox and Mackey. It occurred in summer and was very fatal, but not of the sudden malignant character which has marked the recent cases. It occurred on the flats.

Dr. Greene asked as to the length of time occupied by the former epidemic, the present one had extended over nearly fifteen months.

Dr. Rochester replied that the present one was unusually long continued. As to the period of incubation he did not think that there was any fixed time, certainly nothing could be definitely stated.

Dr. Wyckoff inquired concerning the supposed prophylactic powers of belladonna.

Dr. Rochester replied that he did not believe there was any prophylactic. Influenced by a statement in the earlier edition of Watson's practice he had tested the virtues of belladonna, and for a time thought that it possessed some prophylactic virtues, but he soon found that it did no good. He recited some notable instances in which it had been tested and proved of no avail. Would attend to the clothing, diet and cleanliness of children, but knew of no drug which could be called in any manner a prophylactic.

Dr. White called attention to the fact that in latter editions of his work, Dr. Watson denied the efficacy of belladonna as a prophylactic. He called upon Dr. Greene to recite his experience in his section of the city.

Dr. Greene said that living in a lower section of the city, and practicing on what are called the flats to a considerable degree, he had observed the disease in a very malignant character in the past fifteen months. It had in several instances, which he related, carried away whole families of children. He had observed from

November 1, 1873 to November 1, 1874, 159 cases of which he had a few statistics. Deaths 27. Ratio of deaths 1 in $5\frac{24}{27}$. Died of throat affections, 11; of brain affections, 14; of renal, 2; renal disease manifested itself in seventeen cases. Had observed the disease in ten adults. The oldest patient was fifty, the youngest three months. One woman had scarlatina during confinement, and two children had a second attack. Sequelæ were observed in thirty cases, ten were complicated by diphtheria.

Treatment was based upon general principles; the vaponser was employed in throat affections, using lime water with very pleasant effects. The highest temperature observed was 170°, the thermometer being employed in a large number of cases.

Dr. White referred to the occurrence of cholera in 1832 and 34; as cholera subsided in 1832, scarlatina appeared and was not absent in the inetrim between 1832 and 34. The disease prevailed to a large extent between Black Rock and Lower Black Rock, and in some cases assumed a malignant type.

Dr. Cronyn had seen many cases, several in its most malignant type. In regard to the general features of this epidemic he thought it pursued the course of this disease in former years, at one time very malignnat, at another very mild. Brettonneau formed the opinion during one epidemic that it was not a fatal disease, but upon observing another epidemic a few years afterward was forced to change his opinion.

In 1801–2–3–4 and 5 Ireland was visited especially by a very severe epidemic, robbing almost every household of some of its children. The present epidemic is very prevalent, occurring in Italy, where it is said to have originated some hundred years ago. In London, Dublin, etc. Agreed with Dr. R. as to the general characteristics of this epidemic as far as he had seen it.

Dr. Clifford Albutt had recently written an article on causes of death in first few days, these he enumerated as four hyper-pyrexia, specific poisoning, extreme malignancy, and syncope and asthenia.

In hyper-pyrexia Dr. Albutt used baths, commencing at about the normal temperature and gradually reducing the temperature to about 70°. In a recent case he had put this in practice, the temperature reached 113°, and so violent was the fever that desquammation had twice occurred. He used wet sheet pack. Was very much pleased with result of the treatment. In regard to contagion did not see why there was any difference of opinion. It was undoubtedly a fometic matter which could be conveyed in atmosphere and clothing. In the last few weeks he had noticed a case in which a doctor in Lambeth received a newspaper from Glasgow, announcing the death of his brother's child, in a few days he was taken sick with scarlet fever, and in a short time received a letter from his brother saying that his child had died of scarlatina. Did not believe that there was any definite period of incubation; related several eases of the direct contagion of disease, one in which it occurred during the period of desquamation in the child who conveyed the disease. Therapeutically, he employed the sulphides, in throat complications chlorate of potassa and iron, tonics, etc., in other conditions. Related a recent instance of the trial of belladonna as a prophylactic, in which it had failed—in a naval institute.

Dr. Harrington asked as to giving ice and cold drinks. Dr. C. replied that when the temperature was high he allowed their use, when normal he gave warm drinks when desired.

Dr. Gay was called upon, but being obliged to leave was unable to make any extended remarks, would desire a continuance of the discussion at an adjourned meeting.

Dr. Cronyn spoke as to the relation of scarlet fever, erysepilas and puerperal fever, and said that it was very desirable that the parturient woman should be excluded as far as possible from the danger of contagion.

Dr. Rochester moved that Drs. Elliott, W. C. Cronyn and E. B. Potter, who were present by invitation, be invited to participate. Carried.

Dr. Wyckoff's experience had been some what similar to that of the speakers who had preceded him. Had seen some mild cases and some very malignant. Believed that scarlatina was contagious, also that it originated *de novo*, and cited some instances in which no exposure could be traced in any way. Was governed more by general condition of patient than by any other thing in treatment.

Had seen some cases in which diptheretic complications occurred, in these he had employed iodine in a vaporized form. Had no faith in preventive influence of belladonna, although he knew that there were still some who believed in its efficacy, and had recently seen it again advanced by physicians of some note.

Dr. HARRINGTON asked if scarlatina had been observed in nursing children.

Dr. WYCKOFF replied in the affirmative, and that he had seen nursing children take it first in family, which was contrary to former experience.

Dr. SHAW could date back thirty-three years when an extensive epidemic occurred in Chautaqua county, although he had in the course of his practice seen a large number of cases—could add nothing new.

Dr. LYNDE—Had seen several epidemics. The mode of origin and communication was wholly inexplicable. In Springville, Erie county, he had observed a very severe epidemic. He read a brief resumé of his views.

Dr. HARRINGTON said Aitken affirmed that the disease was three times as fatal in the city as in the country, and in certain localities of his practice he had observed this to be the case; he had observed many more fatal cases in crowded neighborhoods. Had seen one case where a female was attacked in a few days after confinement; had used ice in many cases with very pleasant results to his patients.

Dr. ELLIOTT said that he agreed with the former speakers.

Dr. WHITE called upon the Ex-Health Physician, Dr. O'Brien, and the present incumbent, Dr. Chace, for remarks.

Dr. O'BRIEN could offer nothing in addition to what had been said concerning the character of the disease. The mortality last year was about 630. In December it was 66, in January about 60.

The disease was not confined to this locality alone, but was prevalent in Syracuse, Rochester, Lockport and the surrounding towns in this vicinity. The spread of the disease was aided by those in whose families it was present. Thought that more stringent measures should be employed to prevent communication between the sick and the well.

Keepers of stores and other places liable to be frequented by children, should be compelled to close their places of business when connected with their dwelling on the appearance of scarlatina. The regulations in regard to funerals should be as stringent as in small pox.

Dr. CHACE stated that the Board of Health had been backward in making any regulations from the fact that they lacked a unanimous expression of the profession. He would like very much to have a definite statement by this Association.

Dr. WHITE remarked that he was pleased to observe the entire unanimity existing in the expression of the views of the Association, and would offer the following proposition as expressing in a condensed form the opinion of the members: 1. The terms scarlet fever and scarlatina are synonomous. and are applied to one and the same disease.

2. Scarlet fever is prevailing in the city at present as an epidemic, but it is not unprecedented in severity.

3. It is communicable and can be carried in clothing.

4. Under such circumstances in all individuals who are made susceptible by the epidemic influence, it is developed by individual contact, although occasionally it may arise *de nevo.*

5. Patients should be isolated from the commencement of the attack until the full period of desquamation is passed. The period of incubation is not well settled.

6. Hygienic care in the sick room is of great importance, including ventilation, cleanliness of person and clothing, good food, etc.

7. The secretions of the body should be immediately disinfected.

8. After the termination of a case the room which the patient has occupied should be thoroughly disinfected, ventilated and cleaned.

9. The use of belladonna or other so called preventative medication, is of no value. Keeping the individual in a condition conductive to perfect health is the best means of preventing or mitigating the disease. Impure air, uncleanly and crowded apartments, are liable to add to its severity.

10. Treatment is to be pursued upon general principles. Each individual case is to be treated as the symptoms present themselves. So-called specifics are wholly valueless.

11. It seldom attacks the same person the second time.

12. Great care should be exercised in avoiding undue exposure for sometime subsequent to convalescence, as troublesome and often dangerous sequelæ are apt to follow.

On motion of Dr. SAMO, seconded by Dr. WYCKOFF, this summary was unanimously adopted, and the Secretary instructed to prepare copies for the public press.

The propositions for membership of Drs. Willoughby and Bielby were received, and, under the rules, were laid over for one month.

On motion, the Society adjourned.

